# Screening and brief interventions for hazardous and harmful alcohol use among patients with active tuberculosis attending primary public care clinics in South Africa: results from a cluster randomized controlled trial

**DOI:** 10.1186/1471-2458-13-699

**Published:** 2013-07-31

**Authors:** Karl Peltzer, Pamela Naidoo, Julia Louw, Gladys Matseke, Khangelani Zuma, Gugu Mchunu, Bomkazi Tutshana, Musawenkosi Mabaso

**Affiliations:** 1 HIV, AIDS, TB, and STIs (HAST), Human Sciences Research Council (HSRC), Pretoria, Cape Town, South Africa; 2 HIV, AIDS, TB, and STIs (HAST), Human Sciences Research Council (HSRC), Durban, Cape Town, South Africa; 3 HIV, AIDS, TB, and STIs (HAST), Human Sciences Research Council (HSRC), Port Elizabeth, Cape Town, South Africa; 4 Department of Psychology, University of the Free State, Bloemfontein, South Africa; 5 ASEAN Institute for Health Development, Mahidol University, Salaya, Thailand; 6 Population Health, Health Systems and Innovations (PHHSI), Human Sciences Research Council (HSRC), Cape Town, South Africa; 7 Department of Psychology, University of the Western Cape, Cape Town, South Africa

## Abstract

**Background:**

In 2008 the World Health Organization (WHO) reported that South Africa had the highest tuberculosis (TB) incidence in the world. This high incidence rate is linked to a number of factors, including HIV co-infection and alcohol use disorders. The diagnosis and treatment package for TB and HIV co-infection is relatively well established in South Africa. However, because alcohol use disorders may present more insidiously, making it difficult to diagnose, those patients with active TB and misusing alcohol are not easily cured from TB. With this in mind, the primary purpose of this cluster randomized controlled trial was to provide screening for alcohol misuse and to test the effectiveness of brief interventions in reducing alcohol intake in those patients with active TB found to be misusing alcohol in primary public health care clinics in three districts in South Africa.

**Methods:**

Within each of the three provinces targeted, one district with the highest TB burden was selected. Furthermore, 14 primary health care facilities with the highest TB caseload in each district were selected. In each district, 7 of the 14 (50%) clinics were randomly assigned to a control arm and another 7 of the 14 (50%) clinics assigned to intervention arm. At the clinic level systematic sampling was used to recruit newly diagnosed and retreatment TB patients. Those consenting were screened for alcohol misuse using the Alcohol Use Disorder Identification Test (AUDIT). Patients who screened positive for alcohol misuse over a 6-month period were given either a brief intervention based on the Information-Motivation-Behavioural Skills (IMB) Model or an alcohol use health education leaflet.

**Results:**

Of the 4882 tuberculosis patients screened for alcohol and agreed to participate in the trial, 1196 (24.6%) tested positive for the AUDIT. Among the 853 (71%) patients who also attended the 6-month follow-up session, the frequency of positive screening results at baseline/follow-up were 100/21.2% for the AUDIT (P < 0.001) for the control group and 100/16.8% (P < 0.001) for the intervention group. The intervention effect on the AUDIT score was statistically not significant. The intervention effect was also not significant for hazardous or harmful drinkers and alcohol dependent drinkers (AUDIT: 7–40), alcohol dependent drinkers and heavy episodic drinking, while the control group effect was significant for hazardous drinkers (AUDIT: 7–19) (P = 0.035).

**Conclusion:**

The results suggest that alcohol screening and the provision of a health education leaflet on sensible drinking performed at the beginning of anti-tuberculosis treatment in public primary care settings may be effective in reducing alcohol consumption.

**Trial registrations:**

PACTR201105000297151

## Background

The 2008 World Health Organization (WHO) Report found that South Africa had the highest tuberculosis (TB) incidence in the world, at over 5 times the average incidence rate found in the 22 high-burden countries [1]. In 2006, South Africa with only 0.7% of the world’s population had an estimated 28% of HIV positive adult TB cases reported globally [1]. Over the last 5 years TB case notification has increased by a massive 81%, from 188 695 cases in 2001 to 341 165 cases in 2006 [1]. This increase is mostly associated with the co-morbidity between HIV and TB. However, other factors, such as poverty and alcohol misuse is also associated with TB incidence.

Excessive alcohol use has been causally linked to TB incidence, as alcohol consumption increases the risk of infectious disease, such as TB, considerably. Two pathways are involved: the first is biological via weakening of the immune system, and the second is social via social exclusion and drift, resulting in about a threefold increased risk of TB [2,3]. Alcohol use was estimated to have been responsible for 939 000 disability-adjusted life-years lost in South Africa for TB and HIV/AIDS alone in 2004 (253 000 for women, 687 000 for men) [2]. This figure corresponds to 4.6% of the overall disease burden in South Africa (2.5% for women, 6.6% for men) [2]. These numbers show the potential for reducing alcohol-attributable infectious disease burden in South Africa, since cost-effective measures for reducing alcohol-attributable harm in developing societies exists and could be applied [2].

There are numerous studies cited in the literature that support the strong association between alcohol use, alcohol use disorders and TB [3-8]. Numerous studies show the pathogenic impact of alcohol on the immune system causing susceptibility to TB among drinkers [4,7,9]. “Alcohol use strongly influences both the incidence and the outcome of the disease and was found to be linked to altered pharmacokinetics of medicines used in the treatment of TB, social marginalization and drift, higher rate of re-infection, higher rate of treatment defaults and development of drug-resistant forms of TB; about 10% of the TB cases globally were estimated to be attributable to alcohol” [3], p.1. People that drink heavily show higher relapse rates, a higher probability of an unfavourable clinical course and a higher probability of experiencing the most destructive forms of TB [3,5].

Hazardous drinking is defined as a quantity or pattern of alcohol consumption that places patients at risk for adverse health events, while harmful drinking is defined as alcohol consumption that results in adverse events (e.g., physical or psychological harm) [10]. In studies in South Africa high rates of hazardous or harmful drinking or possible alcohol dependence using the Alcohol Use Disorder Identification Test (AUDIT) were found among tuberculosis patients (23.2%-36%) [11,12]. Previous studies using the same alcohol measure (the AUDIT) found lower rates of hazardous or harmful alcohol use or possible alcohol dependence in general public primary care patients in South Africa 19.2% [13] and 19.2% [14] and in a national population-based survey in South Africa 9% [15].

### Screening and brief interventions for alcohol use disorders

Increasing emphasis has been placed on the detection and treatment of hazardous and harmful drinking disorders, particularly among patients who are seen in primary health care settings [11]. Screening instruments such as the AUDIT, CAGE (Cut Down, Annoyed, Guilty and Eye Opener), and Screening and Brief Interventions (SBIs) have been found to be useful in detecting and treating alcohol use disorders in a number of settings [13,16,17]. The interventions are based on cognitive-behavioural interventions and motivational interviewing techniques and have been found to be effective and efficient in the treatment of alcohol use disorders in most chronic conditions. In previous studies screening and brief intervention for alcohol problems have been successfully implemented by nurses in demonstration projects in South Africa as part of a WHO strategy to expand screening and brief intervention for alcohol problems in developing countries funded by WHO and NIAAA [16,18]. Community health workers have been identified as strategic implementation agents for screening and brief intervention of alcohol problems in primary care in South Africa [16].

Whilst there have been studies conducted on screening for alcohol misuse and brief interventions producing favourable results [19,20], there is a dearth of scientific literature on evidence-based best practice methods to screen for alcohol misuse and brief interventions amongst individuals with active TB [21]. In South Africa the target rate set by the WHO for TB cure has not been met, and the fact that alcohol use and misuse is known to be one of the causal factors in TB onset, there is an urgent need to interrogate behavioural models that address reduction in alcohol consumption. Given that the sample for the current study was from three districts with the highest TB burden in South Africa, reducing alcohol consumption will be valuable in that, it will reduce the participants’ vulnerability to unfavourable TB treatment outcomes, such as treatment failure, a high treatment default rate, TB re-infection and the development of resistant forms of TB. Thus reducing the incidence rate for the population at large.

### Aim of the study

The aim of this study was to conduct a cluster randomized control trial to assess the effectiveness of SBI for alcohol use disorders among TB patients in public primary care clinics. Consenting patients who started TB treatment and screened for risky consumption of alcohol using a standardized tool were randomized, with the public primary care clinic being the unit of randomization into one of two arms: The first arm being a Brief Intervention for alcohol misuse arm (treatment arm) and the second arm being the treatment as usual in addition to receiving an alcohol education leaflet (control arm). We hypothesized that compared to the control group, patients receiving brief alcohol intervention in the intervention group would reduce the overall AUDIT score and would increase screening negative on the AUDIT. The null hypothesis of the study was that the mean AUDIT scores and those screening negative on the AUDIT between those in the intervention and control groups will not be statistically significantly different.

## Methods

### Setting

Three provinces, in South Africa, with the highest TB caseload were selected for inclusion in the study. One district in each province (N = 3) with the highest TB caseloads were consequently included. These districts were Siyanda in the Northern Cape Province, Nelson Mandela Metro in the Eastern Cape Province, and eThekwini in KwaZulu-Natal Province. Within each of these three study districts 14 public primary health care facilities were selected based on the highest TB caseloads per clinic (N = 42). The type of health facility was a primary health care clinic or community health centre. The study catchment areas within the study health districts and randomization procedures enabled a broad coverage of major population groups.

### Design

In order to assess the effectiveness of the Screening and Brief Interventions (SBI) among tuberculosis patients found to be misusing alcohol, a cluster randomized controlled trial design was implemented. All new TB and retreatment patients were screened using the AUDIT. TB patients who met the cut-off for misusing alcohol both in the intervention and control arms were reassessed after baseline assessment at time 2 (3 months following intervention) and time 3 (6 months following intervention). The intervention comprised the following: personalized feedback on AUDIT results, a health education leaflet, simple advice and brief counselling about reducing excessive drinking, during one −20 minute- session. The trial incorporated cluster randomization of public primary health care facilities to avoid the risk of contamination.

### Principles for recruitment

#### Inclusion criteria

##### Public primary health care clinics

Clinics with a high TB caseload (based on statistics provided by the Department of Health) in each of the three study districts with a high burden of TB were included in the study.

##### Patients  with  active  TB

New tuberculosis and retreatment patients (i.e. those patients who have been initiated or have been on anti-TB treatment for less than one month), males and females, 18 years and above who visited the primary health care facility and who scored 8 or more for men and 7 or more for women on the AUDIT questionnaire after the screening were included in this study. A few studies have reported that brief interventions are effective even among excessive drinkers [22-24]. Therefore patients scoring high on the AUDIT were also included in the study. Under 18 years of age were excluded since in South Africa the legal minimum age for alcohol use is 18 years and the study aimed at providing an intervention for hazardous, or harmful drinking or alcohol dependence.

### Randomisation

Randomisation was conducted using a secure remote randomization service. Within each district in each of the three provinces the 14 public primary health clinics with the highest TB caseloads were randomly assigned to the treatment and control arms. A secure remote randomisation service carried out randomisation. Twenty one allocations were initially generated for each of the possible factorial combinations of clinic type, TB case load, and intervention. Randomisation was stratified by clinic type (clinic and community health centre) and TB case load. The standby and reallocated clinics were subsequently randomly allocated in a similar manner. TB patients misusing alcohol in intervention clinics were receiving treatment and those in control clinics received a health education leaflet. At the clinic level all consecutive new and retreatment TB patients were systematically recruited over a period of six months from mid- April to mid- October 2011.

### Blinding

Participants (clinic staff members and TB patients) were not blinded to their intervention or control status. However, to protect against information biases in the reporting of alcohol use and TB treatment adherence behaviour, the data collection team who assessed the outcomes were blinded to the clinic’s status as intervention or control group.

#### Procedure

Universal screening of all new tuberculosis and retreatment patients was used where all consecutive patients visiting the public primary health care facility were screened for alcohol misuse and offered a brief counselling intervention if they met the criteria for misuse of alcohol. A health care provider who identified a new or retreatment TB patient (within one month of treatment initiation) informed the patient about the study and referred the patient for participation if interested. A research assistant asked for consent from patients attending the public primary care facility to participate in stage 1 of the study, i.e. screening or baseline assessment using the AUDIT questionnaire. To increase the reliability of the AUDIT, researchers have suggested locating alcohol consumption within the context of other health-related behaviours [25]. The interviewer administered questionnaire, therefore, included questions on mental and physical health status, tobacco use and chronic diseases. The research assistant was not involved in delivering the brief counselling intervention. All participants underwent the initial assessment and the research assistant scored the results of the alcohol test section of the questionnaire. Tuberculosis patients who scored 8 or more for men and 7 or more for women on the AUDIT questionnaire after the screening (risky drinkers) were then approached by the research assistant for a second informed consent for enrolment in stage 2, the intervention study. For patients included in the study, the research assistant referred the patient to a clinic lay counsellor who carried out the intervention (experimental = brief counselling intervention or control = provision of health education leaflet) for all the participants during the same visit. Thereafter patients were scheduled for follow-up sessions at 3 and 6 months at the health facility, and the follow-up assessments were conducted by the research assistant. The experimental brief intervention included a brief counselling intervention on alcohol risk reduction consisting of two sessions, the first immediately after alcohol screening and the second within a month thereafter. In the control condition the clinic lay counsellor provided an alcohol education leaflet. Sampling occurred throughout all hours of clinic operation over a 6 months period from mid March 2011 to mid October 2011. Research assistants conducted follow-up interviews at 3 and 6 months following baseline assessment at scheduled clinic visits. Several attempts were made to follow-up participants. Firstly, HSRC co-ordinators visited the clinics to access participants fieldwork records with permission of the professional staff in an attempt to get contact details of participants. Secondly, fieldworkers did home visits where no telephone numbers were available for participants. Finally, a special team of research assistants were employed to make direct calls to both fieldworkers and participants to help find participants to schedule appointments. Participants received Rand 60 (8.5 US$) transport reimbursement at 6-month follow-up. Ethical approval was obtained from the Human Sciences Research Council Research Ethics Committee (Protocol REC No.1/16/02/11). The Department of Health in South Africa has also provided approval for this study. Consent to participate was obtained in a 2-stage process. Research assistants initially asked for informed consent to conduct a health screen and collected basic information and checked eligibility to take part. No identifiable information was collected at this stage. Those patients who had a positive score on the AUDIT (alcohol risk score), had the study explained to them verbally and in writing (using the patient information sheet). Informed consent was obtained at this second stage which included permission to give the contact details to the research staff, participate in the experimental or control condition and follow up after 3 and 6 months by the research assistant and provide permission to medical file information.

### Screening and baseline assessment

To identify drinking and other health behaviours, interviews with potential study volunteers were conducted by trained research assistants using a standardized questionnaire. The 10-item AUDIT [23] assessed alcohol consumption level (3 items), symptoms of alcohol dependence (3 items), and problems associated with alcohol use (4 items). Responses to items in the AUDIT were rated on a 4-point Likert scale from 0 to 4, for a maximum score of 40 points. Higher AUDIT scores indicate more severe levels of risk: a score of 8 or more indicates a tendency for problematic drinking, and a score of 20 or above probable alcohol dependence [26]. Heavy episodic drinking is defined as the consumption of six standard drinks (10 g alcohol) or more on a single occasion [26]. In South Africa a standard drink is 12 g alcohol. Because AUDIT is reported to be less sensitive at identifying risk drinking in women [17], the cut-off points of binge drinking for women (4 units) were reduced by one unit as compared with men (5 units), as recommended by Freeborn and others [17]. The AUDIT has been validated in HIV patients in South Africa showing excellent sensi-tivity and specificity in detecting MINI-defined dependence/abuse (sensitivity, 100% and correctly classified 79% of individuals who did not have alcohol abuse or dependence) [27] and among TB and HIV patients in primary care in Zambia demonstrating good discriminatory ability in detecting MINI-defined current AUDs (AUDIT = 0.98 for women and 0.75 for men) [28]. To comply with the timeline of this study, all subjects were asked for their alcohol consumption in the previous 3 months rather than in the past 12 months.

#### Socioeconomic characteristics

A researcher-designed questionnaire was used to record information on participants’ age, gender, educational level, marital status, income, employment status, dwelling characteristics and residential status. *Poverty* was assessed with 5 items on the availability or non-availability of shelter, fuel or electricity, clean water, food and cash income in the past week. Response options ranged from 1 = “Not one day” to 4 = “Every day of the week”. Poverty was defined as higher scores on non-availability of essential items. The total score ranged from 5 to 20, 5 = being low, 6-12 = medium and 13-20 = high poverty. Cronbach alpha for this poverty index was 0.89 in this sample.

#### Tobacco use

Two questions were asked about the use of tobacco products. 1) Do you currently use one or more of the following tobacco products (cigarettes, snuff, chewing tobacco, cigars, etc.)? Response options were “yes” or “no”. 2) In the past month, how often have you used one or more of the following tobacco products (cigarettes, snuff, chewing tobacco, cigars, etc.)? Response options were once or twice, weekly, almost daily and daily.

TB treatment status, HIV status and antiretroviral treatment were assessed by self-report and from medical information. Patients were also asked about a list of chronic and other illness conditions they had been diagnosed with such as diabetes.

### Interventions

#### Health education leaflet

All the patients randomized and allocated to the control group completed the baseline measures and received a health education leaflet on responsible drinking.

#### Brief counselling

The patients randomized and allocated to the intervention arm completed baseline measures and received brief counselling for alcohol reduction intervention. The goals for brief counselling were as follows: 1) To identify any alcohol- related problems mentioned in the interview, 2) To introduce the sensible drinking leaflet, emphasis the idea of sensible drinking limits, and make sure that patients realize that they are in the risk drinking category, 3) To provide feedback on the relationship between alcohol and TB treatment [21,29], 4) To work through the first 3 sections of the problem solving manual while mentioning the value of reviewing the other sections, 5) To describe drinking diary cards, 6) To identify a helper, and 7) To plan a follow-up counselling session. The Information-Motivation-Behavioural Skills (IMB) Model was used in the study to guide the alcohol reduction intervention. The IMB model [30-32] proposes that *information* about alcohol misuse and methods of reducing and preventing harmful and/or hazardous drinking is a necessary precursor to risk reduction. *Motivation* to change, however, also directly affects whether one acts on information about risk and risk reduction. Finally, the IMB model holds that *behavioural skills* related to preventive actions represent a final common pathway for information and motivation to result in alcohol risk behaviour change. The IMB model posits that information and motivation activate behavioural skills to ultimately enact risk reduction behaviours. The IMB model also shows that information or motivation alone can have direct effects on some preventive behaviour, such as when information about risky alcohol drinking prompts drinking at moderate levels or to stop drinking more detail, [33]. The intervention was composed of two scheduled sessions: on Day 1 and within one month after the baseline evaluation. Each session comprised 15–20 minutes of counselling.

### Counsellor training and intervention quality assurance

The intervention counsellors consisted of lay HIV counsellors from the study clinics who spoke the predominant languages, namely English, Afrikaans, i-Zulu, i-Xhosa and Tswana, in the respective areas. These counsellors delivered the interventions to the participants of the study in addition to the “treatment as usual”. The treatment “as usual” consisted of the provision of standardised short-course chemotherapy under direct observation of treatment (DOT) for at least the initial phase of treatment to all identified smear-positive TB cases in all study clinics [34]. All lay counsellors and up to four nurses per study clinic who were suitable to deliver the brief counselling intervention received formal training (lay counsellors 3 days and nurses 2 days) and supervision prior to the start of the study. It was agreed that the HIV lay counsellors would implement the intervention and the trained nurses will assist when necessary. The training took a practical, systems approach, aiming to facilitate the implementation of SBI in clinic operations rather than merely educating staff. The training curriculum contained modules addressing practical issues deemed essential to implementing the programme. For early identification of alcohol problems in public primary care the AUDIT [26] and for the brief intervention the WHO brief intervention package for hazardous and harmful drinking [35] was used. Both were adapted to the South African context, e.g. in terms of standard unit of alcoholic drink and drinking limits. The AUDIT was translated and back translated according to scientific standard procedures [26] into four of the major languages (Tsonga, Northern Sotho, Venda, and Afrikaans) [16,18] and also translated into additional languages (Xhosa, Zulu and Tswana). The self-help booklet for patients and a hand-out on “cutting back” showing the drinking limits and health effects of risky alcohol consumption were also made available in the languages relevant for the study. The AUDIT manual explains the purpose of screening for alcohol problems in primary care, the context of alcohol screening, the development and validation of the AUDIT, administration guidelines, scoring and interpretation. The Brief Intervention manual defines concepts and terms, roles and responsibilities of Public Primary Health Care, SBI: a risk management and case finding approach, alcohol education for low-risk drinkers, abstainers and others, and simple advice a brief counselling for risk zone drinkers, self-help booklet and training sources. Critical administrative activities included administration and scoring of the screening instruments, assuring availability of patient brochures, sequencing of interventions with treatment of presenting health problems, the essential elements of an intervention, and the management of SBI records.

The training comprised of four elements: orientation to the relevant practice, standardised power point presentation, tape recorded simulated consultations with trained actors and on-going clinical supervision by experienced HSRC staff. The simulated consultations were recorded and rated by two independent clinical assessors. The brief intervention counsellor was assessed for adherence to the brief counselling protocol in addition to their behaviour and skills using a Behaviour Change Counselling Index [36].

Assessors submitted the ratings, comments and supervision points for each consultation. This information supported clinical supervision and training until the brief intervention counsellor reached a required standard of practice agreed by the assessors [37].

To help protect against counsellor drift (deviating from the existing treatment intervention protocol), the brief intervention was completely manualized and was used to guide the counsellor through the content of the session. Lay counsellors received bi-weekly support visits by the project trainers during the implementation of the project. Monthly visits were paid by research staff to the clinics in support for the project to assist them with any technical aspects of the counselling intervention. In terms of control around the quality and consistency of the implementation of the intervention, intervention counsellors filled in a patient monitoring form for each counselling session conducted. It included sections on the client’s AUDIT score, stage of change, action and intervention plan, handing of action plan to client and comments. A readiness ladder was used to assess a patients’ readiness to change their drinking, i.e. their stage of change. Patients were asked to rate on a ladder from ‘not at all’, ‘a little’, ‘somewhat’, ‘very’ to ‘extremely’, “How important is it for you to change your drinking?” Patients who score in the lower end (‘not at all’) of the scale were classified as pre-contemplators, those who score in the middle range (‘a little’ – ‘somewhat’) as contemplators, and those scoring in the higher range were considered as ready to take action. Intervention counsellors were compensated with R20 (US$2.6) for each counselling session conducted and monitoring form completed. In addition, study fieldworkers were able to report to their coordinators regarding any problems they may be having in implementing the brief interventions. Regular meetings between the researchers and the project managers allowed for any problems to be resolved timeously.

#### Outcome evaluation

A health survey questionnaire was administered in a face-to-face interview in scheduled appointments at the clinic was used to collect outcome information at 3 and 6 months following the baseline assessment. In addition, medical file information was collected for HIV and TB treatment status and outcome. Non-attenders were followed up by telephone and home visits arranged as necessary. The primary outcome was the (1) change in the mean score on the AUDIT in the last 3 months and the number of AUDIT negative drinkers in the last month of the study period compared with baseline, as measured by the AUDIT. The secondary outcome was the successful TB response, classified by WHO as cured or treatment completed (versus treatment failure, defaulted, died or transferred out to another health facility [38].

### Sample size calculation

Using the Practihc Trial Protocol tool sample size is calculated with cluster size as a binary outcome based on an average cluster size of 350, an intra-cluster correlation coefficient of 0.2, with 90% power and 5% significance level. A previous review [39] found a clinically important difference in negative status on AUDIT between brief intervention and controls of 13% (5% reduction in controls and 18% in brief intervention recipients). With an expected 20% AUDIT negative in the intervention and 5% in the control, a minimum of 200 sample per group (total 400), with 20 clusters per group, would be needed. Assuming a loss to follow-up of 40%, the sample size was inflated to 280 per group (total 560). As initially only new TB patients were considered in the trial but later new retreatment patients were also included in the trial the sample size was doubled to 1120.

### Data management and analyses

Data were captured by dedicated data capturers. Data cleaning and data verification was supervised by key project staff, including the principal investigator and methodologist. The verification process included double data entry of all questionnaires and its fields, doing programmed range checks by computer to identify outlying values, checking for missing values, and checking for inconsistencies in the data. Cluster-specific methods of data analysis (Generalized Estimating Equations) were used because we randomized clinics rather than patients. Data were analysed using STATA Version 11.

#### Intention to treat

The principle of the statistical analysis was intention to treat. It was applied to the two hierarchical levels of the trial: the clinics randomized at the cluster level and the patients recruited within each of these units.

##### Clinic level

In all 36 units randomized were included in the analysis. Units were analysed according to the intervention group allocated at randomization. The stratification used for the randomization of clinics (TB caseload and type of facility) were taken into account in the analysis.

##### Patient level

In all 853 patients recruited to the study were used in the analysis of the primary and secondary outcomes. Patients were analysed as a member of the clinic in which he/she was recruited.

#### Analysis approach

The primary outcome was measured at three time points: baseline, three and at six months. If a patient dropped out, and is not present on the day of the interview or refuses to answer questions the primary outcome at the end point of the trial was missing. Therefore, except for the baseline measurement, no post-randomization information was available for these participants. The extent of the missing component was 29% at six months. The method used to take account of the stratified cluster trial design, the repeated binary and linear nature of the primary and secondary outcome (Risky drinking, TB treatment outcome) and the missing data at follow-up is a generalized estimation equations (GEE) approach [40]. The total AUDIT score had to be log-transformed [x’ = log(x + 1)] beforehand, with the aim of making the distribution less skewed. The logarithmically transformed AUDIT score was compared between groups by Student *t* test. Because the randomization had been on clinic level, and to correct for baseline differences between the two groups, multilevel logistic regression was performed for binominal, and multilevel linear regression for continuous variables. Estimated treatment effects are reported with 95% confidence intervals. Descriptive statistics were calculated for baseline and follow-up.

## Results

### Screening and randomization

**Figure 1:**
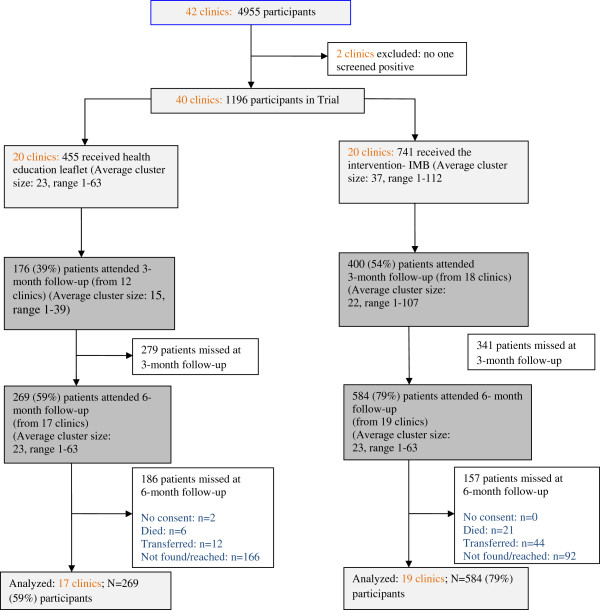
Flow-chart of clinics and participants in the trial.

Figure 1 summarizes patient identification, recruitment, randomization, and follow-up numbers. We identified 4955 public primary care TB patients of which 3684 screened negative for alcohol, 51 refused to participate and 24 were found ineligible, resulting in 1196 patients across 40 primary care clinics. Of the 4880 screened for alcohol and agreed to participate in the trial 1196 (24.6%) tested positive for the AUDIT. Participants in clinics were randomized into 20 control and 20 intervention clinics using the clinic as a unit of randomization to 455 patients in the control group and 741 in the intervention group. As illustrated in Figure 1, response rates were higher in the intervention than in the control group at both follow-ups. At the 3-month follow-up, response rates for the control and intervention were 39% and 54%, respectively, and at 6 months, the control and intervention group response rates were 59% and 79%, respectively. In the control group 41% did not complete the last follow-up survey (i.e., the dropout rate was 41%); in the intervention group, 21% did not complete the last follow-up survey. The main difference between groups was in the dropout rate (percentage of participants that did not complete the last follow-up survey): 21% in the intervention group versus 41% in the control group.

Attrition analyses were conducted to check for differential attrition by examining the condition by dropout interactions at baseline. Dropout was significantly related to the condition (P < 0.001) and poverty (P = 0.003), indicating a relationship between these variables and dropout status depending on condition: 21% in the intervention group versus 41% in the control group dropped out and the poverty index was 9.9 in the intervention versus 8.9 in the control group. Dropout was not related to gender (P = 0.308), age (P = 0.078), education (P = 0.866), AUDIT score (P = 0.958), AUDIT (7–40) (P = 0.516), AUDIT (7–19) (P = 0.952), AUDIT (20–40) (P = 0.918), heavy episodic drinking (P = 0.192), daily or almost daily tobacco use (P = 0.929), HIV status (P = 0.488), and TB new or retreatment patient (P = 0.082).

### Brief intervention implementation fidelity analysis

Assessment of intervention fidelity via the patient monitoring forms found that the intervention was delivered with sufficient fidelity. In 75% of the intervention sessions, the lay counsellors implemented at least 6 of the 7 requisite intervention steps (including, e.g., to describe drinking diary cards, stage of change, action and intervention plan). In addition, it was found that in 96% of the cases of brief intervention, only one session was conducted despite having scheduled a follow-up session, and in 4% of cases two sessions. Further analysis of the monitoring form assessing the stage of change in the intervention group found that 7.4% were at the precontemplation, 38.9% at the contemplation, 34% preparation and 19.7% at the action stage.

### Participant characteristics

Table 1 summarizes demographic, health variables and alcohol-related characteristics of the study participants. Overall, the study sample was 74.3% male, averaged 36.7 years of age, 19.3% had grade 12 or more education and 21.8% scored high on the poverty index. With regard to health variables, 19.9% were TB retreatment and 80.1% new TB patients, 54.2% were HIV positive, 73.2% were high risk drinkers and 26.8% were probable alcohol dependent. The mean AUDIT score was 15.7 (see Table 1).

**Table 1:** Baseline descriptive information

**Variables**	**Control**	**Intervention**
	**N = 455 (%)**	**N = 741 (%)**
**Socio-demographic variables**		
Gender (N,% male)	328 (73.4)	545 (74.9)
Age (M, SD)	35.9 (10.8)	37.2 (11.0)
*Education*		
Grade 7 or less	134 (29.9)	251 (34.5)
Grade 8-11	237 (52.5)	329 (45.2)
Grade 12 or more	79 (17.5)	148 (20.3)
*Poverty index (5–20)*		
Low (5)	134 (31.0)	191 (28.2)
Medium (6–12)	235 (54.4)	307 (45.3)
High (13–20)	63 (14.6)	179 (26.4)
**Health variables**		
*Perceived health status*		
Excellent	47 (10.4)	55 (7.5)
Very good	68 (15.0)	60 (8.2)
Good	133 (29.4)	282 (38.4)
Fair	130 (28.8)	254 (34.6)
Poor	74 (16.4)	83 (11.3)
AUDIT total (M,SD)	14.2 (6.0)	16.5 (6.5)
AUDIT (7–19)	369 (80.7)	507 (68.6)
AUDIT (20–40)	88 (19.3)	232 (31.4)
New TB patient	344 (78.0)	592 (81.3)
Retreatment TB patient	97 (22.0)	136 (18.7)
Daily or almost daily tobacco use	171 (38.1)	267 (39.8)
HIV positive	210 (46.5)	435 (59.0)
HIV negative	215 (47.6)	245 (33.2)
HIV unknown	27 (6.0)	56 (7.6)
On antiretroviral therapy	91 (29.8)	142 (32.3)

### Drinking and TB treatment outcomes

There were significant reductions in the AUDIT score (AUDIT total score, hazardous or harmful drinking or alcohol dependence, hazardous drinking, alcohol dependence and heavy episodic drinking) over time across treatment groups. Among the 853 (71%) patients who also attended the 6-month follow-up session, the frequency of positive screening results at baseline/follow-up were100/ 21.2% for the AUDIT (P < 0.001) for the control group and 100/16.8% (P < 0.001) for the intervention group. The intervention effect on the AUDIT score was statistically not significant. The intervention effect was also not significant for hazardous or harmful drinkers and alcohol dependent drinkers (AUDIT: 7–40), alcohol dependent drinkers and heavy episodic drinking, while the control group effect was significant for hazardous drinkers (AUDIT: 7–19) (P = 0.035). Further, there was also no significant intervention effect for daily or almost daily tobacco users and at 6-month follow-up the intervention group did not significantly differ to the control group in terms of TB treatment cure or completion rate (see Table 2).

**Table 2:** Alcohol-related outcome measures at baseline, 3-month and 6-month follow-up and TB treatment outcome at 6-month follow-up

**Variables**	**Time**	**Control**	**Intervention**	OR* (95% CI)	**P-value**	**ICC (SE)**
				**Adjusted for cluster**		
AUDIT total (M,SD)	Baseline	14.2 (6.1)	16.5 (6.5)	0.91 (0.78-1.07)	0.264	0.13 (0.04)
	3 months	4.0 (5.9)	5.0 (6.1)			
	6 months	3.6 (6.2)	2.4 (4.8)			
AUDIT (7–40) High risk or alcohol dependence	Baseline	455 (100)	741 (100)	0.70 (0.41-1.19)	0.186	0.11 (0.03)
	3 months	37 (21.0)	139 (34.8)			
	6 months	57 (21.2)	98 (16.8)			
AUDIT (7–19) High risk (N,%)	Baseline	367 (80.7)	507 (68.4)	0.64 (0.43-0.97)	0.035	0.07 (0.02)
	3 months	33 (18.8)	126 (31.5)			
	6 months	45 (16.7)	91 (15.6)			
AUDIT (20–40) Alcohol dependence (N,%)	Baseline	88 (19.3)	233 (31.4)	1.39 (0.75-2.56)	0.296	0.09 (0.03)
	3 months	4 (2.3)	13 (3.2)			
	6 months	12 (4.5)	7 (1.2)			
Heavy episodic drinking^1^ (weekly+) (N,%)	Baseline	131 (29.0)	331 (45.2)	0.96 (0.46-2.02)	0.921	0.22 (0.06)
	3 months	11 (9.6)	23 (10.4)			
	6 months	13 (11.6)	16 (10.6)			
Daily or almost daily tobacco use (N,%)	Baseline	171 (39.0)	278 (40.8)	1.12 (0.67-1.89)	0.662	0.09 (0.03)
	6 months	71 (31.8)	93 (18.6)			
*TB treatment outcomes (N,%)*^2^	6 months	195 (53.6)	289 (45.9)			
Cure		76 (20.9)	135 (21.5)			
Complete		22 (6.0)	29 (4.6)			
Failure		65 (17.9)	155 (24.6)			
Default		6 (1.6)	21 (3.3)			
Died		12 (3.2)	44 (6.5)			
Transfer out						
TB treatment cure or completion	6 months	271 (74.5)	424 (67.4)	0.93 (0.46-1.88)	0.840	0.15 (0.5)

^1^For men 5 or more and for women 4 or more drinks on one occasion.

^2^ TB treatment outcomes defined as from WHO classification [35] was not complete (N = 1049; 88%) due to various reasons including missing codebook of fieldworker lost, the incorrect recording of names, names could not be found in clinic register, and clinic register lost.

*OR* Odds Ratio, *ICC* Intracluster correlation coefficient, *SE* Standard Error.

*Odds ratios from regression models were adjusted for age, sex, and baseline alcohol use disorders identification test score.

## Discussion

To our knowledge, this is the first randomized trial to evaluate the effectiveness of a brief intervention for hazardous and harmful drinkers with tuberculosis in public primary care clinics in South Africa. The trial was conducted in 40 public primary care clinics including sites in urban and rural areas in three different districts in three different provinces in South Africa with a high TB disease burden. These diverse settings strengthen the generalizability of the findings.

Self-reported outcome data suggest that the provision of a health education leaflet can help reduce levels of hazardous and harmful alcohol use in those TB patients attending public primary care in South Africa. From baseline to 3- and 6-month follow-up, alcohol consumption declined significantly in both intervention and control groups. The intervention effect was, however, not statistically significant on the AUDIT score, hazardous or harmful drinkers and alcohol dependent drinkers (AUDIT: 7–40), alcohol dependent drinkers and heavy episodic drinking, while the control group effect was significant for hazardous drinkers (AUDIT: 7–19).

The significant reduction of hazardous or harmful alcohol use and possible alcohol dependence found in our trial in the control or no-treatment group has at least three possible explanations, including 1) the intervention effect of alcohol screening/follow-up and provision of health education leaflet on sensible alcohol drinking, 2) the intervention effect of standard care (nurses provide advice on alcohol drinking) and 3) natural history changes in drinking over time in the course of TB treatment. McCambridge and Kypri [41] reviewed that simply answering questions on drinking in brief intervention trials appears to alter subsequent self-reported behaviour. This potentially generates a bias by exposing non-intervention control groups to an integral component of the intervention. The effects of brief alcohol interventions may thus have been consistently under-estimated. Based on the assessment of stage of change in alcohol drinking by the lay counsellors in the intervention group, it was found that already a large proportion of the patients (53.7%) have been in the preparation or action stage of change, according to the IMB model. In addition, qualitative information collected in a sub-sample of follow-up interviews seemed to confirm the reduction of alcohol use due to the health care provider’s advice and due to illness and TB treatment condition. Other studies e.g., [42] seem to have shown a reduction of alcohol use with the initiation of ART, which could be similar with the initiation to TB treatment.

Further, the study found that there was no significant difference in TB treatment cure or completion rate between intervention and control group at 6 months follow-up. It is possible that there are factors, such as enabling or disabling individual, familial and community-level conditions, which were not taken into account nor measured in the study. In addition, the study did not find a significant reduction of tobacco use in the intervention compared to the control group, which is in line with a recent review [43] that found brief alcohol interventions do not also reduce cigarette smoking, and it appears unlikely that there exist other important secondary effects.

Studies of brief intervention delivery in primary care by lay counsellors are much rarer than by health care professionals. Lay counsellor delivered brief alcohol interventions with TB patients seems to be feasible to implement with fidelity in the South African primary care setting, as found in the context of HIV risk reduction counselling in South Africa [44].

### Study limitations

Our study has several limitations, including the loss of patients at each follow-up point. Despite randomization there were baseline differences between the two groups on several covariate measures. This may be due to the relatively large variation of the sample size on the level of group assignment. Although we controlled for these differences, we cannot exclude that there are additional unmeasured baseline differences that confound the effect, a fact that reduces internal validity of the study. The high dropout rate in the control compared to the intervention group represents a threat to the validity of the findings of this study and the most likely source of bias. Further, alcohol use was only assessed by self-report. The consensus in the research community that self-reported alcohol consumption was valid derives mainly from conclusions drawn from studies undertaken in treatment contexts [45]. It is not clear whether influences on the validity of self-report may be different in South Africa. Bias in alcohol consumption may have resulted from self-reported outcome measures. Future studies should consider assessing alcohol consumption by both self-report and objective measures, such as blood alcohol level. Further, the study only assessed short-term intervention effects (6 months) and longer term assessments (12 months) would be needed, especially because TB patients after having been cured may start drinking again.

## Conclusion

In this rigorously conducted trial, we succeeded in implementing a lay counsellor led brief alcohol inter-vention in a general clinic-based sample of hazardous and harmful drinkers. The short duration of the brief intervention makes it a realistic candidate for use in primary health care. Based on this study evidence the effectiveness of brief interventions in TB public primary care patients is still inconclusive. The reduced alcohol consumption of the control group may have resulted from the screening assessment at baseline, the provision of the health education leaflet on sensible drinking and the natural history of alcohol misuse in the course of TB treatment. More studies are needed to explore the effects of brief alcohol interventions with tuberculosis patients in primary care settings. The results suggest that alcohol screening and the provision of a health education leaflet on sensible drinking performed at the beginning of anti-tuberculosis treatment in public primary care settings may be effective in reducing alcohol consumption.

## Abbreviations

AUDIT: Alcohol use disorders identification test; CAGE: Cut down annoyed guilty and eye opener (alcohol use disorders screening test); IMB: Information-motivation-behavioural skills model; NIAAA: National institute on alcohol abuse and alcoholism; SBI: Screening and brief intervention; TB: Tuberculosis; WHO: World Health Organization.

## Competing interests

The authors declare that they have no competing interests.

## Authors’ contributions

KP, PN and GM were the main contributors to the conceptualization of the study. KP, PN, JL and GM contributed significantly to the first draft of the paper and all authors contributed to the subsequent drafts and finalization. All authors read and approved the final manuscript.

## Pre-publication history

The pre-publication history for this paper can be accessed here:

http://www.biomedcentral.com/1471-2458/13/699/prepub
